# The Stop Signal Task for Measuring Behavioral Inhibition in Mice With Increased Sensitivity and High-Throughput Operation

**DOI:** 10.3389/fnbeh.2021.777767

**Published:** 2021-12-09

**Authors:** Alican Caglayan, Katharina Stumpenhorst, York Winter

**Affiliations:** ^1^Institute for Biology, Humboldt University, Berlin, Germany; ^2^Excellenzcluster NeuroCure, Charité Universitätsmedizin Berlin, Berlin, Germany

**Keywords:** stop signal task, sorting system, behavioral inhibition, mouse models, automated behavioral analysis

## Abstract

Ceasing an ongoing motor response requires action cancelation. This is impaired in many pathologies such as attention deficit disorder and schizophrenia. Action cancelation is measured by the stop signal task that estimates how quickly a motor response can be stopped when it is already being executed. Apart from human studies, the stop signal task has been used to investigate neurobiological mechanisms of action cancelation overwhelmingly in rats and only rarely in mice, despite the need for a genetic model approach. Contributing factors to the limited number of mice studies may be the long and laborious training that is necessary and the requirement for a very loud (100 dB) stop signal. We overcame these limitations by employing a fully automated home-cage-based setup. We connected a home-cage to the operant box via a gating mechanism, that allowed individual ID chipped mice to start sessions voluntarily. Furthermore, we added a negative reinforcement consisting of a mild air puff with escape option to the protocol. This specifically improved baseline inhibition to 94% (from 84% with the conventional approach). To measure baseline inhibition the stop is signaled immediately with trial onset thus measuring action restraint rather than action cancelation ability. A high baseline allowed us to measure action cancelation ability with higher sensitivity. Furthermore, our setup allowed us to reduce the intensity of the acoustic stop signal from 100 to 70 dB. We constructed inhibition curves from stop trials with daily adjusted delays to estimate stop signal reaction times (SSRTs). SSRTs (median 88 ms) were lower than reported previously, which we attribute to the observed high baseline inhibition. Our automated training protocol reduced training time by 17% while also promoting minimal experimenter involvement. This sensitive and labor efficient stop signal task procedure should therefore facilitate the investigation of action cancelation pathologies in genetic mouse models.

## Introduction

Inhibition of a pre-potent response, usually when the response is no longer appropriate, is defined as behavioral inhibition (Wong, 2013). Action cancelation is a specific type of inhibition where an ongoing motor response is stopped while it is already being executed. The stop signal task (SST) was developed to measure this ability of action cancelation (Logan et al., 1984; Tannock et al., 1989). In this task, subjects are first trained to perform a fast reaction with a defined beginning and end, the so called go response following a go signal. In the stop signal task with mice this “go signal” corresponds to an initial nose poke which triggers a light signal and immediately continues into a rapid motor action that ends in a second nose poke to a neighboring nose poke hole.

After a go response has been established, a stop signal is introduced in some of the trials (e.g., 20%). It informs the subject to stop its ongoing motor response such that after the first nose poke the second nose poke is suppressed. The stop signal is given with some delay (stop signal delay, SSD) after the first nose poke initiates the motor response. The longer this delay the harder it is for a subject to respond to the stop signal and inhibit its ongoing motor response.

Deficits in action cancelation ability occur in many psychiatric and neurological conditions such as attention deficit and hyperactivity disorder (Schachar et al., 2000; Overtoom et al., 2002; Alderson et al., 2007), schizophrenia (Hughes et al., 2012; Yang et al., 2020), obsessive-compulsive disorder (McLaughlin et al., 2016; Mancini et al., 2018), Parkinson’s disease (Gauggel et al., 2004; Obeso et al., 2011) and substance abuse (Monterosso et al., 2005; Li et al., 2006; Zhang et al., 2018). To understand the contribution of action cancelation deficits to those pathological conditions and develop better treatment, animal models are crucial. Furthermore, the stop signal task is crucial for dissociating the neurobiological mechanisms of action cancelation and action restraint (Johnstone et al., 2007; Eagle et al., 2008; Dambacher et al., 2014).

The stop signal task, first introduced for rats two decades ago (Feola et al., 2000), has been used to investigate: (i) anatomical structures and neurotransmitter systems involved in action cancelation (Eagle and Robbins, 2003a,b; Eagle et al., 2007; Bari et al., 2009, 2011), (ii) neural activity correlates of action cancelation (Bryden et al., 2012; Leventhal et al., 2012; Schmidt et al., 2013; Mayse et al., 2015; Mallet et al., 2016), and (iii) cognitive aspects of action cancelation (Beuk et al., 2014; Mayse et al., 2014; Xu et al., 2019). The stop signal task for mice (Humby et al., 2013) has been used to identify genetic correlates of action cancelation (Davies et al., 2014, 2019; Dent et al., 2016). Considering that psychiatric conditions with an impairment in action cancelation such as attention deficit and hyperactivity disorder (Thapar and Cooper, 2016; Faraone and Larsson, 2019) and schizophrenia (Cardno and Gottesman, 2000; Sullivan et al., 2003) have a significant genetic component, studies using this task with genetic mouse models have remained surprisingly scarce.

One of the factors that have limited the use of genetic mouse models with the stop signal task is the labor requirement due to the task’s long training period (∼44 days, Humby et al., 2013). In general, labor requirement can be reduced by utilizing automated home-cage based experimentation, that allows continuous experimentation with minimal experimenter involvement (Endo et al., 2011; Schaefers and Winter, 2011; Balci et al., 2013; Remmelink et al., 2016, 2017). Moreover, such minimized experimenter involvement can also reduce data variability (Crabbe et al., 1999; Sorge et al., 2014), and lead to more consistent results across laboratories (Lipp et al., 2005; Krackow et al., 2010).

We previously developed an ID chip based gating mechanism for home-cage based experimentation (Winter and Schaefers, 2011; Caglayan et al., 2021). This allows continuous testing with self-initiated individual experimental sessions by letting only one individual at a time into the experimental compartment. Therefore, individuals are free from interference by cage mates during sessions. Moreover, this gating mechanism allows setting individual inter-session intervals so that a recently admitted individual cannot reenter. Longer intersession intervals (e.g., 1 h) ensure high engagement during a time-restricted session (e.g., 30 min). Rodents readily adapt to the gating mechanism and the sorting procedure and have been shown to perform various operant tasks (Schaefers and Winter, 2011; Winter and Schaefers, 2011; Rivalan et al., 2017; Qiao et al., 2018; Caglayan et al., 2021). In this study, we employed a gating mechanism as one component of a more efficient stop signal experimental procedure for mice.

Another factor that limits the use of mice in the stop signal task is the requirement for a rather loud stop signal (100 dB, 300 ms white noise) to train mice (Humby et al., 2013). It necessitates placing the operant chamber in a sound-attenuating box, which requires considerable space. Furthermore, despite the use of these relatively loud stop signals in previous studies behavioral inhibition observed in mice has remained relatively low (∼85%) (Humby et al., 2013; Davies et al., 2014) on stop signal baseline trials, i.e., trials during which the stop signal is given simultaneously with the “go.” During these baseline trials (without stop signal delay), the task becomes a go/no-go task and measures action restraint rather than action cancelation (Eagle et al., 2008). As action restraint ability will also affect the outcomes during trials with stop signal delays, any inability for action restraint will confound action cancelation measurements. A lower level of baseline inhibition therefore limits the sensitivity of the test as it reduces the measurable outcome for action cancelation from 0–100% to for example 0–85% behavioral inhibition. Such ceiling or floor effects are shown to affect the observed effect sizes (Šimkovic and Träuble, 2019) and in turn can lead to decreased sensitivity. Moreover, variability in action restraint ability will add to the observed variability of the outcome measurements. Increased variability will decrease the sensitivity of a test. Furthermore, as stop signal reaction times are calculated based on the probability of behavioral inhibition, the resulting estimates will be more accurate the smaller the influence from action restrain. Hence, achieving relatively high baseline inhibition with low variance can increase the sensitivity and accuracy of the stop signal task. We were able to greatly improve baseline inhibition by introducing a mild air puff as a negative reinforcer, as described in previous go/no-go tasks (Harrison et al., 2016; Chen et al., 2019; Maor et al., 2020).

With this study we established an automated stop signal task for group housed mice that is based on a gating mechanism for individual separation and uses a mild air puff with escape option to enhance operant conditioning. We estimated individual stop signal reaction times (SSRT) and show that mice can be trained to a high level of baseline inhibition in the stop signal task.

## Materials and Methods

### Animals

Twelve C57BL/6JRj female mice (Charles River, Germany) aged 8 weeks were housed in groups of six in standard EU type III cages (43 × 27 × 18cm). Animals were kept on a 12 h light/12 h dark cycle at 23 ± 2°C and 45–55% rel. humidity. For identification in the holding facility mice had received biocompatible subcutaneous ID chips prior to this study. Experiments were carried out with two groups of six animals in succession. Water and maintenance chow (V1535, Ssniff, Germany) was provided *ad libitum* until the start of the experiment. During experiments mice obtained all of their food from the reward dispenser within the operant chamber. Food intake and weight were monitored daily. Due to the reward pellets’ high caloric content, mice generally maintained their free-feeding weight. On very rare occasions when a mouse obtained less than 50 pellets per day it was provided with 1 h of free feeding with maintenance chow in a separate cage. One mouse was excluded due to task disengagement following the introduction of a stop signal and another one due to health problems (mouth infection).

### Ethics

All procedures were conducted in compliance with the European Communities Council Directive 2010/63/EU and under the supervision and with the approval of the animal welfare officer at Humboldt University. Due to the study’s observational nature, the animals were free from damage, pain or suffering. Our undisturbed home-cage-based approach aims to obtain data while maximizing animal welfare.

### Apparatus

**FIGURE 1:**
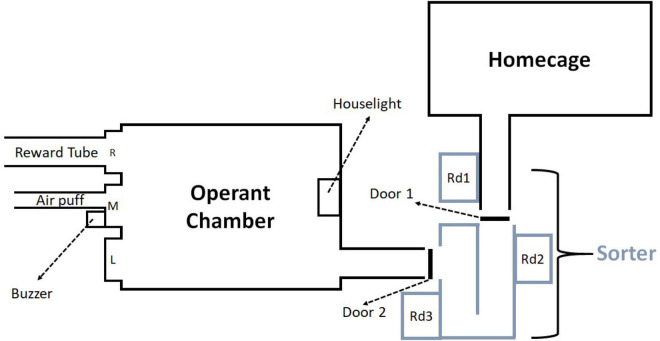
The automated dual cage system consisted of three parts: a home cage, a sorter and an operant chamber. The sorter connected the operant chamber and home cage. When reader (Rd) 1 detected a mouse door 1 opened, and when the mouse had proceeded to reader 3 door 1 closed. Both doors remained closed for 30 s to verify that only a single mouse was inside the sorter. Afterward, door 2 opened and the mouse proceeded to the operant chamber for a session (30 min). The operant chamber contained three ports with nose-poke sensors (L: left, M: middle, R: right), a 4.5 kHz buzzer used for the stop signal and a house light to signal timeout. L and M were used as response ports, R was used as a reward port. A metal tube within M could deliver air puffs to negatively reinforce responses after a stop signal. A pellet feeder delivered pellets to R.

The experimental system (Figure 1) consisted of a home cage, an RFID-based mouse sorter (ID Sorter, PhenoSys) and an operant chamber (Med Associates, ENV-307A). The software PhenoSoft Control (PhenoSys) controlled all components of the system from a PC. The operating principles of the sorter have been described previously (Winter and Schaefers, 2011). Briefly, the sorter consists of a U-shaped tunnel with a guillotine door at each end and three ID chip sensors. The sorter connected the home-cage with the operant box via Plexiglas tubes. When a mouse was detected at reader 1, door 1 opened and the mouse could walk into the sorter. Once the mouse was detected at reader 3, door 1 closed. Thereafter the mouse stayed within the sorter for 30 s. During this 30 s interval data from reader 2 and reader 3 was used to verify that only one mouse was inside the sorter before door 2 opened, and the mouse could enter the operant chamber for a session (30 min). The sorter remained closed until the mouse completed the session. Afterward it returned through the sorter to the home cage. The operant chamber contained three ports equipped with nose poke detectors. Two functioned as response ports (left and middle) and the third as a pellet reward receptacle (right). A buzzer provided the stop signal (4.5 kHz, 70 dB), and a house light signaled timeout. A custom-made air puff (middle response port) negatively reinforced “false alarm” responses, i.e., nose poke responses despite a stop signal (for details, see Supplementary Materials and Methods).

### Behavioral Procedure

**FIGURE 2:**
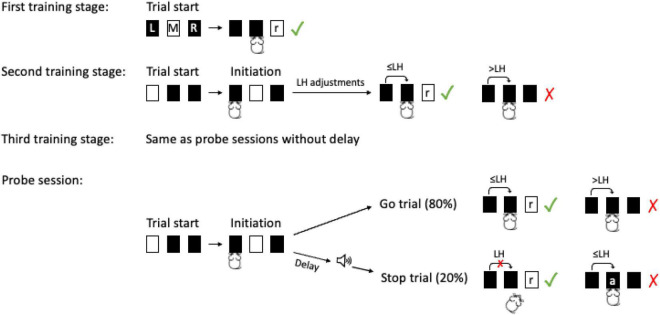
Stop signal task training and testing stages. First training stage: mice had to perform a nose to the illuminated response port (M) to receive a reward (r) from the right port (R). There was no time limit for this response. Second training stage: Upon trial start mice had to poke the initiation port (L). There was no time limit for this response. After the initiation poke mice had to poke M within a limited hold duration (LH) to successfully complete the go response and receive a reward. At this stage LHs were individually adjusted to train fast go responses. Third training stage: a stop signal was presented in 20% of trials immediately upon poke onset to the initiation port. In stop trials, a poke to the response port within LH was negatively reinforced by an air puff (a). If instead, the mouse suppressed the poke to the response port it was rewarded with a pellet after the LH. Probe sessions: Delays between the poke to the initiation port and the stop signal were introduced. The delays were individually set relative to an individual‘s mean reaction time from the previous day (75, 150, and 300 ms before the mean reaction time). In each stop trial, there was an equal probability (0.33) to receive one the three different delay durations (mouse icon from Selman Design, CC BY).

Besides habituation to the operant box and sorter, we followed a three-stage training procedure (Figure 2) to teach mice the following steps: (i) inserting the head into the left port to initiate trials (first training stage), (ii) performing fast go responses by quickly nose poking the middle port after trial initiation (second training stage) and, (iii) stopping this go response upon a stop signal while at the same time maintaining fast and reliable go responses on trials without stop signal (third training stage).

Mice were not time limited to initiate trials. However, they had a time limit to complete go responses (limited hold, LH) once a trial had been initiated. During the second training stage, the limited hold was gradually decreased to ensure a fast go response. Failing to complete a go response after trial initiation (omission) was negatively reinforced by a 45 s timeout (go trial timeout). To increase motivation sugar pellets (TestDiet, 5TUN, 14 mg) were added with a ratio of one in three to the purified pellets (TestDiet, 5TUL, 14 mg) during this stage. In stage three, the stop signal was introduced to 20% of trials (randomly chosen). At this training stage, the stop signal was presented immediately upon trial initiation. Completion of the go response after a stop signal led to negative reinforcement with an air puff (∼1 bar, 20 ms) which was given after a short delay (air puff delay, 200 ms), and was followed by a timeout (45 s, stop trial timeout). To achieve fast responding with a high completion rate at go trials (>70%) and high behavioral inhibition at stop trials (> 85%), we adjusted the experimental parameters limited hold, go trial timeout, and air puff delay for each mouse individually (Supplementary Materials and Methods, Supplementary Tables 1, 2 for individual parameters of each stage and final values). After training was completed, mice proceeded to probe sessions.

Probe sessions were administered for 10 days for each mouse. During probe sessions, stop signal delays were introduced between the initiation response (poke port L) and the acoustic stop signal. These stop signal delays were individually determined according to an individual’s previous day’s mean reaction time (Tannock et al., 1989, p. 480). They were set such, that the acoustic stop signal was given 75, 150, or 300 ms before the peak of the individual reaction time distribution (mean reaction time). The order of the delays was random and there was equal probability (0.33) to receive any of these three stop signal delays during a stop signal trial. As the delays were set relative to the mean reaction time, they can also be thought of as “advance notice intervals” before the expected completion of the go response. If a mouse completed the go response by poking port M before the intended stop signal had been given (early trial), it was treated as a regular go trial for the animals and the mouse was rewarded. However, for the analysis these early trials were included as data points in the stop signal reaction time calculations (as a non-inhibited response) and also the inhibition curve (Mayse et al., 2014). According to the reasoning of the two-horse race model (see Supplementary Materials and Methods), very fast responses are part of the reaction time distribution. Therefore, they are also members of the non-inhibited trials distribution and thus must be included. If early trials were excluded this would affect the stop signal reaction time calculation especially if a subject has a high standard deviation of its go reaction times (see theoretical calculations,^1^ Supplementary Results, Supplementary Figure 1).

### Pilot Experiment

Before we introduced an air puff, we tried to establish the task without negative reinforcer using six female mice (same supplier, sex and age as described above). Training stages one and two where the same as described above. However, as we were trying to find the optimal variable settings for mice to learn not to respond after a stop signal, variable settings differed during the third training stage. Independent of these settings we observed low levels and high variability of baseline behavioral inhibition during 28 days of stage three training prior to the introduction of the air puff. Addition of the negative reinforcer increased baseline behavioral response inhibition and reduced variability while mean reaction times and percentages of completed go trials were only slightly affected (for further information see Supplementary Results, Supplementary Figure 2).

### Data Analysis

To obtain inhibition curves and calculate stop signal reaction times (SSRTs), we pooled the data from all probe sessions and counted the number of inhibited and non-inhibited stop trials for each delay. Delays were taken as 75, 150, or 300 ms before the grand mean reaction time of all go responses, irrespective of daily stop signal delay adjustments. Two additional analyses, in which (a) the number of trials per day (i.e., per dynamically adjusted individual reaction time) was taken into account, and (b) inhibition curves and SSRTs were first calculated for each day and then averaged across days led to very similar results (Supplementary Materials and Methods and also Supplementary Results, Supplementary Figures 6, 7).

As mice from time to time did not complete an initiated go trial (omission), the same behavior is likely to have also occurred in stop trials. Therefore, the recorded inhibited probe trials reflect both voluntary response inhibition and omissions. We corrected the number of inhibited trials according to the formula from Tannock et al. (1989) and Solanto et al. (2001) (for further details, see Supplementary Materials and Methods).

To analyze inhibition curves, we used a logistic generalized linear mixed model (GLMM, with logit link and binomial error distribution) and fitted inhibited trials (corrected) and non-inhibited trials as dependent factors, SSD as independent factor, and set a random intercept for each individual mouse. Before fitting the model, SSD was normalized to ensure model convergence (which does not affect the result of the model). We evaluated the z-statistic for statistical significance and also calculated confidence bands around the inhibition curve. Data analysis and visualization were performed using R 4.0.2 (R Core Team, 2020). Confidence bands were calculated by using the ggeffects package (Lüdecke, 2018).

SSRTs were calculated using the two horse race model (Logan, 1994). A distribution of go reaction times during the probe sessions was created, using all three delays. Our inclusion of trials from all delays differs from some previous studies that only used the stop signal trials with delays leading to ∼50% inhibition (Eagle and Robbins, 2003b; Humby et al., 2013). Since we had a high number of go reaction times for each mouse from our 10 days of probe trial sessions we also included the delay “300 ms before mRT” with ∼83% inhibition. Afterward, we averaged the estimates from all three delays to calculate SSRT for individual mice (for further details, see Supplementary Materials and Methods).

### Comparison With Theoretical Inhibition Curves

To examine how well the observed inhibition values matched with theoretical predictions from the two horse race model, we constructed theoretical inhibition curves based on the two horse race model and based on the parameters obtained from experimental data and then compared observed with theoretical inhibition (see Supplementary Materials and Methods). For the theoretical inhibition curves, we assumed a normal distribution of go reaction times although the observed reaction time distributions were slightly right-skewed (Supplementary Results, Supplementary Figure 3). The individual mean reaction times and standard deviations used for theoretical calculations are depicted in Supplementary Materials and Methods, Supplementary Table 3.

## Results

### Sorter and Task Training Results

During the first day of habituation, the sorter was inactive and all doors were open thus providing a simple tunnel connection between operant- and home cage. Mice entered the operant cage and collected an average of 261 pellets per individual from the reward receptacle.

From the second day of habituation onward, the doors of the sorter were active. During the training phases, mice entered the operant cage for five sessions per day on average (less entries at training onset, more, during later stages). Before a sorting procedure was successful a mouse entered the sorter about five times. This was due to incidences of crowding with cage mates within the sorter tube which slows down the sorting procedure.

**FIGURE 3:**
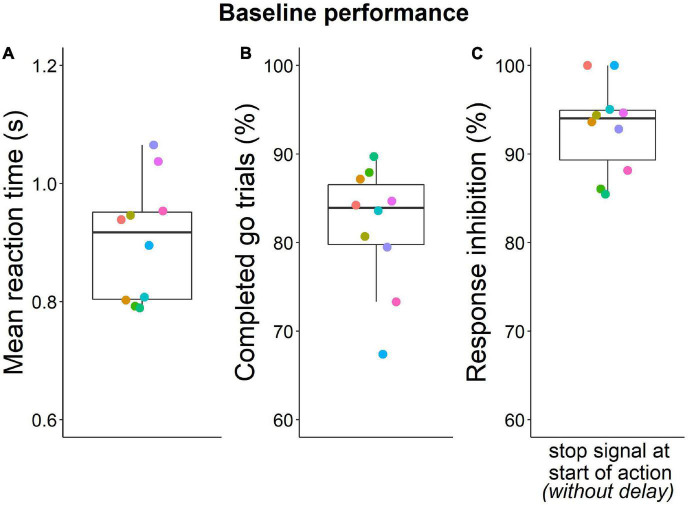
Baseline performance during last training day. **(A)** Mean go response reaction times (median = 0.92 s). **(B)** Percentage of successfully completed go trials (median = 84%). **(C)** Inhibition performance during baseline stop trials (no stop signal delay) (median = 94%). Individuals are represented by the same color in all panels. Box plots show median, 1st and 3rd quartile, and whiskers the 1.5 interquartile range. Data from *n* = 10 mice.

**FIGURE 4:**
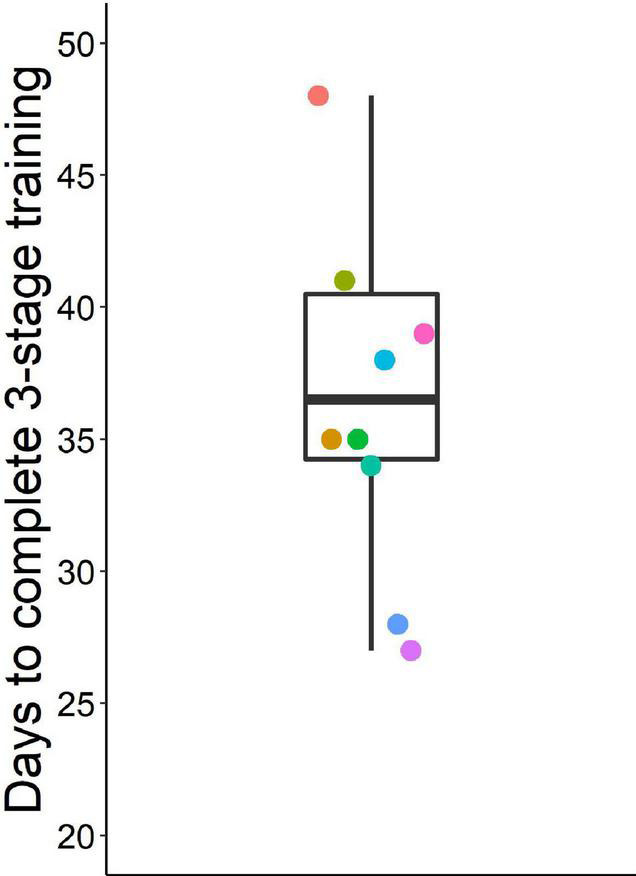
Days to complete three training stages before starting probe sessions. Box plot shows median, 1st and 3rd quartile, and whiskers the 1.5 interquartile range. Data from *n* = 10 mice, a data point at 76 days is not shown.

With the three-stage training procedure, mice eventually learned to quickly (< 1.65 s) make the second poke to the middle port in > 70% of go trials (except one mouse with only 67% go trial performance after extensive training) and to inhibit this second nose poke in 85% of stop trials (Figure 3). On the last day of training, mice completed five to nine sessions (median 6.5) with 110 to 280 trials (median 200) (Supplementary Results, Supplementary Figure 4). Overall, mice required 27 to 76 days (median = 36.5) for training until probe trials began (Figure 4).

### Results From Probe Sessions

**FIGURE 5:**
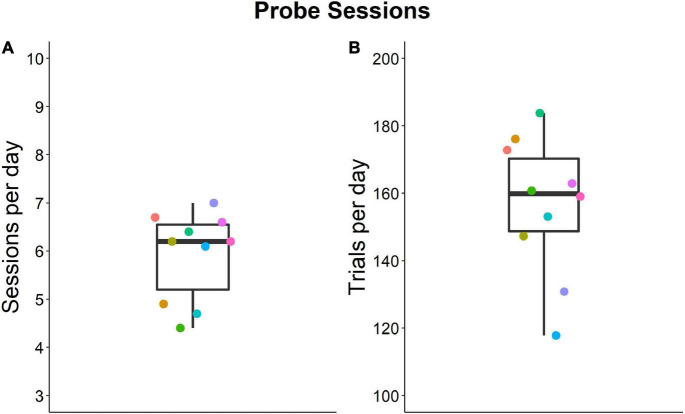
Numbers of individual sessions and trials per day during the probe session phase of the experiment. Individual data are means. Group median values are 6.2 **(A)** and 160 **(B)**. Colors show the same individual. Box plots show median, 1st and 3rd quartile, and whiskers the 1.5 interquartile range. Data from *n* = 10 mice.

Mice completed 4.4–7.0 daily probe sessions each with an average of 118–184 trials per day (Figure 5).

**FIGURE 6:**
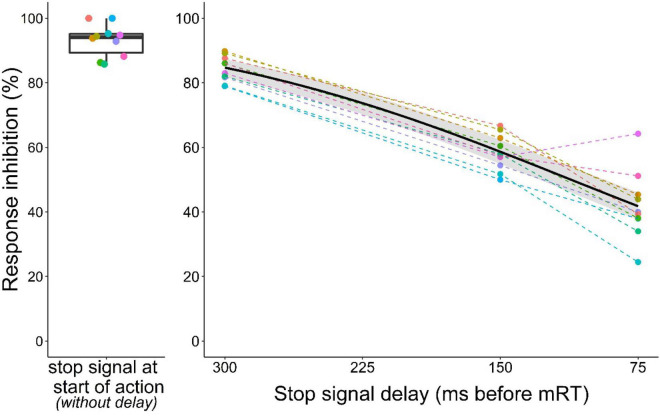
Inhibition curve of mice in the stop signal task. The left panel shows baseline performance (i.e., no delay between trial initiation and stop signal, data from Figure 2C). On the right panel stop signal delays are shown on a scale relative to each individual mean reaction time (mRT). Response inhibition became more difficult the further a mouse had already progressed toward making its second nose poke. Black curve represents the logistic model with 95% confidence interval in gray. Colors identify individuals. Data from *n* = 10 mice.

To determine the effect of the stop signal delay on action cancelation, we fitted a logistic regression model to the data from all stop trials (with stop signal delays 75, 150, and 300 ms before the mean reaction time). As expected with increasing stop signal delays (i.e., less time to process the stop signal), the probability of inhibiting the ongoing motor response decreased (Figure 6, GLMM: *z* = –17.4, *p* < 0.001). Furthermore, comparison of the observed inhibition values with theoretical inhibition curves constructed for each animal based on the two horse race model showed a reasonable fit (Supplementary Results, Supplementary Figure 5).

**FIGURE 7:**
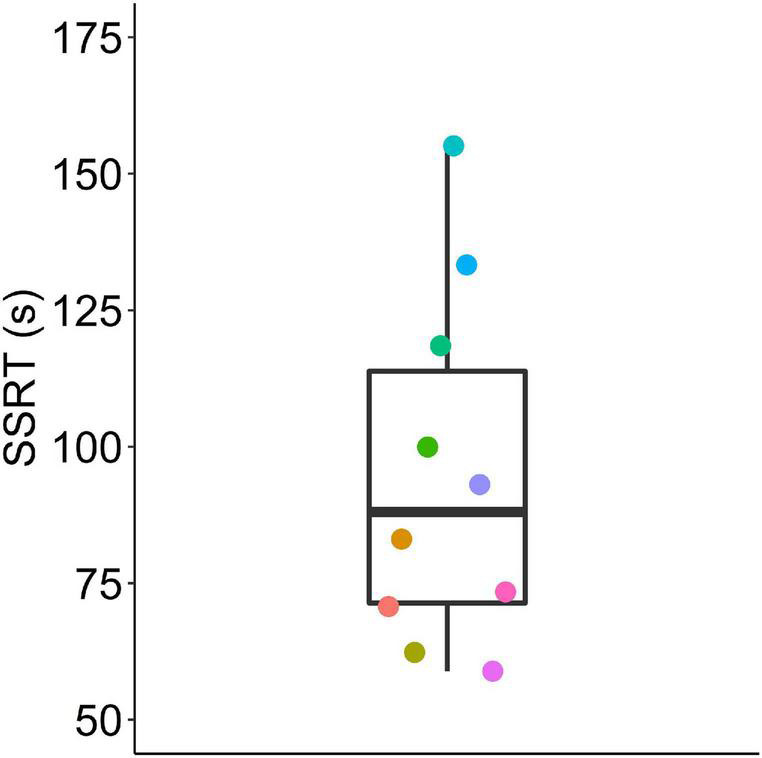
Stop signal reaction times (SSRTs) calculated from pooled data using the two horse race model. Box plot shows median, 1st and 3rd quartile, and whiskers the 1.5 interquartile range. Data from *n* = 10 mice.

We estimated the stop signal reaction time from the pooled data by averaging the calculated SSRTs for each individual from all SSDs (Figure 7). Stop signal reaction times ranged from 59 to 155 ms (with a median of 88).

Two different procedures of constructing inhibition curves and calculating mean SSRTs outlined in Supplementary Materials and Methods led to very similar results (Supplementary Results, Supplementary Figures 6, 7).

## Discussion

The results of this study show that it is possible to greatly improve both the sensitivity and the animal throughput of the mouse stop signal task. This should greatly increase the usability of the SST procedure with mice. Our modifications aimed at improving two aspects of the experiment: Implementing automated procedures made the SST both less labor intensive for experimenters and faster to complete for mice. Introducing a negative reinforcer significantly improved the SST test procedure by greatly increasing the sensitivity of the test. In the following, we shall address this second point first.

The usefulness of any diagnostic procedure depends among others on its sensitivity to the parameter of interest. In the stop signal task this parameter is the stop signal reaction time that is the time required to process the stop signal and cancel the ongoing action. The stop signal is given before the expected time of the completion of the go response as “advance notice interval” and the shorter this becomes (equivalent to a longer delay since action onset) the more difficult it is to respond to it by stopping the action. Estimating SSRT from behavioral performance during tasks critically rests on the assumption that a subject will always stop after processing the stop signal. This is because analysis cannot easily distinguish between the inability of the animal to respond to the stop signal and an animal ignoring the stop signal. Therefore, it is especially important that subjects respond at a high rate to the stop signal in this task. This action restraint ability is trained in baseline trials.

Recognizing that the inability for action restraint can confound action cancelation measurements, initial studies with rats included a correction for baseline stopping errors (Eagle and Robbins, 2003a,b). However, later studies (Eagle et al., 2007; Bari et al., 2009, 2011, 2015) have not followed that example, maybe because rats showed a generally higher level of baseline inhibition. On the other hand, in mice, baseline behavioral inhibition only reached levels around 85% (even with a very loud stop signal) in previous studies (Humby et al., 2013; Davies et al., 2014). In the present study, we increased the baseline inhibition of mice in stop signal trials to an average of 94%, a significant increase over the 84% reported previously (especially considering that, in contrast to previous studies, the baseline inhibition was corrected for omission rate). The major factor responsible for this high degree of task compliance was our air puff negative reinforcer. While air puffs have been used commonly with auditory go/nogo procedures (Otazu et al., 2009; Hangya et al., 2015; Harrison et al., 2016; Chen et al., 2019; Christensen et al., 2019; Maor et al., 2020), our use of an air puff negative reinforcer is novel for the SST. Failing to inhibit the operant response on a stop signal trial commonly only results in to an unrewarded trial and a timeout, thereby reducing the overall probability of obtaining rewards. The introduction of a mild air puff adds an additional aversive dimension to the task procedure, potentially motivating animals to be more attentive to stop signals. In addition, the air puff might have improved the animal‘s ability to separate go trial omissions due to responses after LH from incorrect responses following a stop signal, as otherwise both would have been indicated by a timeout (signaled by a house light). The resulting high baseline inhibition levels result in more precise SSRT and inhibition curve estimates. Moreover, they increase the overall sensitivity of the test as it broadens the range of performances in which experimental groups can vary from each other and thus also prevents a floor effect which could otherwise occur with longer stop signal delays.

In addition, training all mice to high baseline inhibition levels, decreases the variability in baseline inhibition within a group which would otherwise add to the variability of the response inhibition when assessing action cancelation ability. Before we introduced an air puff as a negative reinforcer, we observed high levels of variability in baseline inhibition during a pilot study (Supplementary Results, Supplementary Figure 2). Furthermore, addition of an air puff also decreased baseline inhibition variability compared to a previous study in mice. The standard deviation of baseline inhibition was 8.9 in the conventional setup (Humby et al., 2013, calculated by using the standard error of mean reported in Supplementary Material), while the standard deviation of baseline inhibition in the present study was 5. The introduction of a negative reinforcer thus improved action cancelation assessment in the stop signal task in mice.

In addition, we were able to reduce the sound intensity of the acoustic stop signal from 100 dB commonly used in previous studies (Humby et al., 2013; Davies et al., 2014) to 70 dB. Apart from animal welfare concerns this was especially important for us, as our home cage based systems did not include sound attenuating cabinets. While mice could hear the stop signal in their home cage, rodents are known to be capable of learning the context of auditory signals (Honey and Good, 1993; de Hoz and Nelken, 2014). Similarly, mice learnt auditory cues in an automated home-cage-based auditory discrimination cage, despite multiple such cages being present in the same room (Francis and Kanold, 2017).

After the mice had been trained on the stop signal task, they proceeded to the probe sessions. We used the probe session data to construct inhibition curves. Overall, successful behavioral inhibition decreased with increasing SSD as expected (Logan, 1994), indicating successful SST implementation. Different from earlier authors we analyzed data by fitting a logistic model (GLMM) instead of using ANOVA since the dependent variable was binary (inhibited vs. non-inhibited) and the two horse race model predicts an S-shaped inhibition curve.

Here, we briefly recount the two horse race model which underlies analysis. This model assumes that the go response and the inhibition of that response are two independent processes running concurrently. During a stop trial, a subject may either complete its go response or stop. Stopping occurs when the response inhibition process is completed before the ongoing go response. Both processes are assumed to have a normal distribution and the inhibition process is shorter than the go process. Therefore, by gradually increasing a stop signal delay this leaves less and less time for successful inhibition. The stop signal reaction time SSRT is then inferred from the go reaction time distribution of go trials and the probability of inhibition with a given stop signal delay (Logan et al., 1984; Band et al., 2003; Verbruggen and Logan, 2009).

After constructing the inhibition curve, we estimated SSRTs for each individual using the two horse race model. The median SSRT was 88 ms and therefore considerably lower than ∼350 ms (Humby et al., 2013). However, previously reported SSRTs for rodents have varied widely from ∼150 ms (Mayse et al., 2014) to ∼350 ms (Eagle and Robbins, 2003b), and even within the same study, SSRTs from one batch to another varied significantly (Eagle and Robbins, 2003b, 298 vs. 342 ms in different batches). Therefore, natural variability between batches might contribute to some of the difference between our and previous findings of SSRT values. In macaques, SSRTs estimated from saccades (eye movements) are in a range similar to our results (60–140 ms, Hanes et al., 1998).

We would like to address one other aspect of our study that may have facilitated shorter SSRTs. When compared to previous studies our overall mean reaction times were longer (921 ms compared to 837 ms in Eagle and Robbins, 2003a and 656 ms in Humby et al., 2013). Two factors are likely to have contributed to longer reaction times. First, our experimental apparatus was different. A previous study with mice used a 5-hole chamber where nose poke sensors are directly behind the hole opening and triggered by a shallow poke. Our chamber had three regular food receptacles which required full head insertion to trigger the photo gate. Also, food receptacles were farther apart than holes on a 5-hole wall. The different geometry led to different movement kinematics which needed more time. Equally important may be a second factor that was a consequence of not putting the mice on food restriction. Without food restriction our mice may have been less motivated to perform the task without omissions (Mai et al., 2012; Burnett et al., 2016). As a consequence, we as experimenters, had to adjust the limited hold time interval to a duration that kept experimental omission rate below 30%. With mice that were less motivated our limited hold time interval was therefore most likely longer than it would have been with mice under food restriction. This experimental condition gave mice more time, and allowed mice to move less fast. This chain of reasoning has a further consequence. Slower movement might have eliminated the so-called “ballistic component” of the go response. The “ballistic component” corresponds to the part of the go response that cannot be stopped (e.g., due to muscle activation) even though the stop signal is processed (Logan, 1994; Verbruggen and Logan, 2009). In our case, with slower movement, this “ballistic component” effect may have been reduced thus enabling the mice to stop even on short notice. A corollary of this effect is that we also obtained a relatively high standard deviation and coefficient of variation for pooled go reaction times (mean: 921 ms, *SD*: 200 ms, CV: 21.7%) compared to a previous rat study (mean: 837 ms, SD: 106 ms, CV: 12.7%) (Eagle and Robbins, 2003a). The estimated standard deviations for individual animals ranged between 140 and 213 ms in our study. In a future study a kinematic analysis of stop signal responding using e.g., DeepLabCut (Mathis and Mathis, 2020) may be useful to further improve the sensitivity and resolution of this diagnostic procedure.

Putting rodents on food restriction is common practice to increase performance levels in operant choice experiments with food rewards (Mai et al., 2012; Burnett et al., 2016). Although food restriction played no part in the present study, the potential effect of food restriction increasing motivation and subsequently resulting in more reliable estimates of SSRT should be further investigated. However, even if there is such an effect for probe trials, we show that there is no need to impose food restriction through all training stages. We demonstrated that mice reliably learn the go response and to stop upon a stop signal without any food restriction. If food restriction is to result in more precise SSRT estimates, this food restriction can be imposed at a very late stage of the training just before probe sessions.

In future, the sensitivity of our task can be further validated by using manipulations previously known to affect SSRTs. One commonly used manipulation with SST is lesioning the medial prefrontal cortex (mPFC, Eagle and Robbins, 2003a; Humby et al., 2013). Lesions in mPFC cause impairments in action cancelation similar to humans suffering from cortical dysfunction (Rubia et al., 1999; Robbins, 2007). One can also examine the effects of enhancement with ADHD drugs in both healthy and lesioned animals. Drugs used for ADHD medication (methylphenidate and atomoxetine) are known to improve action cancelation in rodents (Eagle and Robbins, 2003b; Humby et al., 2013) akin to the improvements observed in human patients (Aron et al., 2003; Chamberlain et al., 2007). With similar interventions the predictive and construct validity of our SST can be corroborated.

In addition to higher sensitivity through higher baseline inhibition, our home cage based procedure has the following benefits: (i) a higher level of both animal welfare and experimenter efficiency due to the omission of food restriction and minimized contact between animals and experimenter (ii) individual sessions free from cage mate interference due to the temporary separation of individuals via the sorter, and (iii) reduction in the duration of the training phase of the experiment %17 (median 36.5 days with our system vs ∼44 days in Humby et al., 2013 using the same strain of mice). Other benefits of using a sorter or gating system for home-cage experimentation have been discussed previously (Schaefers and Winter, 2011; Winter and Schaefers, 2011; Rivalan et al., 2017; Caglayan et al., 2021). The additional benefits make our procedure of performing the stop signal task a convenient and efficient tool for studying deficits in action cancelation. Our procedure should greatly facilitate such investigations in mice and genetic mouse models which will remain crucial to investigate the genetic correlates and etiology of action cancelation deficits under pathological conditions such as ADHD, schizophrenia, and OCD.

## Conclusion

Our newly developed mouse stop signal task procedure led to a significantly higher level of compliance by the animals and therefore to higher baseline inhibition. We achieved this by introducing an air puff as a negative reinforcer during stop signal trials. This approach solved a problem of the SST in which mice show a tendency to not restrain their actions despite their general ability to do so and subsequently led to higher task sensitivity. The inhibition curves we obtained from mice confirm that our newly developed procedure yields the expected measure of action cancelation ability (with longer delays, the action is harder to cancel). Furthermore, our procedure of continuous, automated experimentation allowed high throughput under conditions without food restriction, without extensive experimenter involvement and without loud auditory signals. These improvements should facilitate a wider application of the mouse stop signal task, especially important for investigating correlates of action cancelation impairment in gene manipulated mouse models.

## Data Availability Statement

The raw data supporting the conclusions of this article will be made available by the authors, without undue reservation.

## Ethics Statement

The animal study was reviewed and approved by the animal welfare officer of Humboldt University.

## Author Contributions

AC and YW conceived the project. AC conducted the experiment under the supervision of YW and KS. AC performed data analysis and prepared the figures (except Figure 2 was prepared by KS). All authors contributed to writing of the manuscript.

## Conflict of Interest

YW owns PhenoSys equity. The remaining authors declare that the research was conducted in the absence of any commercial or financial relationships that could be construed as a potential conflict of interest.

## Publisher’s Note

All claims expressed in this article are solely those of the authors and do not necessarily represent those of their affiliated organizations, or those of the publisher, the editors and the reviewers. Any product that may be evaluated in this article, or claim that may be made by its manufacturer, is not guaranteed or endorsed by the publisher.

## Footnotes

1
https://zenodo.org/record/5213902#.YSKp1Evit9N
