# Supplementary material for: The Stop Signal Task for Measuring Behavioral Inhibition in Mice With Increased Sensitivity and High-Throughput Operation
Source: Front Behav Neurosci. 2021 Dec 9;15:777767. doi: 10.3389/fnbeh.2021.777767 (PMC8696275; doi:10.3389/fnbeh.2021.777767)
Supplement: Supplementary file 1 [file Data_Sheet_1.docx]

# **Supplementary material and methods**

## **Apparatus**

Our system consisted of three parts: A home cage (EU type III, 43x27x18cm), an RFID-based sorter (ID Sorter, PhenoSys), and an operant chamber (Med Associates, ENV-307A). Experiments with this combined system were controlled by Phenosoft Control (PhenoSys) using the DIG 704 driver (SOF-732-3, MedAssociates) to control the MedAssociates interface box. After a mouse had entered the operant chamber for an experimental session (30 min), the sorter did not allow the passage of any other mouse from the home cage, until the mouse occupying the operant chamber had finished its session and had returned to the sorter.

The operant chamber contained a house light (Med Associates, ENV-315M-LED) to signal timeout and three receptacles (Med Associates, ENV-200R2M) on the opposite wall. Two receptacles were used as response ports (left and middle) and one as the reward port (right). The reward port was connected to a pellet dispenser (Med Associates, ENV-203-20). A buzzer (Med Associates, ENV-323HAW, 4.5 kHz) above the middle port was used to deliver the stop signal (70 dB). Sound intensity was calibrated using a sound level meter (Labmatrix Manufacturing LLP). For air puff delivery a syringe needle (Sterican, G 26 x 1" / Ø 0.45 mm, brown) was shortened and inserted through a drilled hole at the back to protrude approx. 5mm into the middle port. Air delivery was controlled by a valve (Clippart, EV-3M, 24 VDC) with a valve booster (Clippart, EVB-3) using Festo tubing (Festo, PUN, Ø 4 mm, wall thickness: 0.75 mm). Pressurized air (~1 bar) was obtained from a local air compressor situated in another room. A nose poke response into the middle port despite a stop signal resulted in the delivery of an air puff (20 ms).

## **Behavioral procedure**

### **Initial System Habituation**

### During the first day of system habituation, the sorter was inactive and functioned as a walk-through tunnel between the home cage and the operant box. The home cage was connected to the sorter, and mice were free to move between the home cage and the operant box. The reward port (right port) was illuminated by a LED and a reward pellet (TestDiet, 5TUL, 14 mg) was delivered. Upon a nose poke to the reward port, the LED was switched off for 20 s (inter-trial interval, ITI). At the end of the ITI the reward port was illuminated again, and another reward pellet was delivered. The following day, the sorter was active, and mice individually entered the operant box for 30 min sessions by passing through the sorting procedure. The sorting procedure stayed active for the rest of the experiment.

### **First Training-Stage - Single Nose poke responses**

### During the first training stage the mice learned to respond to the middle port, which was illuminated at the start of a session. Upon a nose poke to the middle port the LED went off, the right reward port was illuminated, and a pellet was delivered. After the mouse collected the reward the right LED was switched off and the absence of any illuminated response ports signaled the ITI (1 s). The ITI remained set at 1 s for the rest of the experiment. Following the ITI, the middle port was illuminated again, and a new trial began.

### **Second Training-Stage – Go response**

### During the second training stage mice learned the go response. At the beginning of a trial the left port was illuminated. A nose poke into the left port switched off the LED and triggered illumination of the middle port. The mouse then had a limited time-window (limited hold) to make a nose poke into the middle port to subsequently be rewarded at the right port. If a mouse failed to make a nose poke to the middle port within the limited hold duration, a timeout started (go trial time out, 45 s, signaled by the house light). Limited hold durations were initially set to 30 s and were decreased following a performance-based adjustment. If a mouse responded successfully within the limited hold period in 16 out of a block of 20 trials (i.e. omission rate < 20%), the duration was decreased in steps by 15 s, 10 s, 2.5 s, and 500 ms. Once a limited hold duration reached 2 s, it was further decreased in steps of 100 ms. If the performance of a mouse did not allow a further decrease for a full day, the last limited hold duration was fixed, except if the animal had received less than 50 pellets in which case the limited hold duration was increased by 200ms before being fixed. This approach was chosen to ensure that mice, that responded unusually fast in a 20 trials block, would not remain on a limited hold duration that would prevent them from regularly gaining rewards.

### After all mice in a batch had reached stable, minimal limited hold durations using normal pellets, a 2:1 mixture of normal pellets (TestDiet, 5TUL, 14 mg) and sugar pellets (TestDiet, 5TUL, 14 mg) was placed in the pellet dispenser to further increase motivation. Limited hold durations were then further decreased in 50 ms steps. The criterion for these steps was an omission rate <10% in a block of 30 trials. If a mouse – after the first day of training with sugar pellets - could not further decrease its limited hold within any of the following days, it was fixed for this stage. After all mice had reached a fixed limited hold, they proceeded to the next stage.

### **Third Training-Stage – Action Cancellation while Maintaining a Fast and Reliable Go Response**

In stage three, stop trials were introduced: A stop signal (4.5 kHz, 70 dB) was randomly given in 20% of trials. At this stage the stop signal was given immediately upon nose poke detection at the left port. After a stop signal, mice initially had to refrain from responding to the middle port for only 400 ms before the LED in the middle port was switched off, the reward port was illuminated, and a reward was delivered. This refrain period was raised to 800 ms the following day, and finally it was set equal to the limited hold duration of each individual animal. If a mouse made a response to the middle port during the refrain period, an air puff (~1 bar, 20 ms) was delivered as a negative reinforcer to the middle port, no reward was given and a 45 s timeout (stop trial time out) was started. During the first ten days of the third training stage, the stop signal duration (duration of buzzer) was 400 ms so that mice can learn the stop signal easily. Then the duration was reduced to 150 ms.

In contrast, during go trials, omission to respond to the middle port within the limited hold period continued to result in a go trial time out. However, during the third training-stage the go trial timeout was set according to the individual’s limited hold: For LH ≤ 1.4 s, the go timeout was set to 90 s, for LH > 1.4 s and LH ≤ 1.6 s, it was set to 150 s, and for LH ≥ 1.6 s, it was set to 210 s. With these adjustments to the go trial timeout, mice maintained their fast go responses while also learning to stop following a stop signal. The limited hold period for a mouse continued to be increased during this stage, if the mouse’s omission rate was higher than 30% across all go trials in a day, albeit only in 50 ms steps.

Air puffs were initially delivered with a delay of 200 ms (air puff delay) to enable mice to retract their head and thus avoid the experience. If mice showed less than 85% inhibition across all stop trials of a day, the air puff delay was decreased by 50 ms the next day. One mouse had difficulties keeping its inhibition above 85% for two consecutive days, even when the air puff was given without any delay. For this mouse the air puff duration was in addition increased to 80 ms and the stop signal timeout was increased to 550 s (instead of 20 ms and 45 s, respectively). Under these conditions the mouse showed stable performances with inhibition rates on stop trials above 85% for two consecutive days, though its omission rate on go trials remained with 33% slightly above the criterion of ≤ 30%.

After all mice in the batch had reached the criterion of ≥85% response inhibition in stop trials and ≥ 70% completed go trials for two consecutive days, the parameters were fixed, and the mice proceeded to the probe sessions. Table S1 summarizes the experimental parameters of each stage and Table S2 shows the final parameters that were set for probe sessions for each individual mouse.

| **Table S1.** Training stages. | | | | | |
| --- | --- | --- | --- | --- | --- |
| Stage | Aim | Limited hold | Go trial timeout | Stop trial  timeout | Puff delay |
| First training stage | Initiation response | - | - | - | - |
| Second training stage | Fast go response | Start 30 s  Final 1-1.6 s | 45 s | - |  |
| Third training stage | Stopping upon stop signal | Final 1.3-1.65 s | 90 -210 s (depending on LH) | 45 s (except one mouse) | Start 200 ms  Final 50-200 ms^*^ |
| ^*^For one mouse (Ind9) puff delay had to be decreased to 0 ms, puff duration increased to 80 ms. Furthermore for this mouse the stop signal timeout was also increased to 550 s while it was 45 s for all other mice. | | | | | |

| **Table S2.** Final experimental parameters for each individual at the beginning of probe sessions | | | | | |  |
| --- | --- | --- | --- | --- | --- | --- |
| Individual | Limited Hold  [s] | Go trial timeout  [s] | Stop trial timeout  [s] | Puff Delay  [ms] | |  |
| Ind1 | 1.65 | 210 | 45 |  | 50 | |
| Ind2 | 1.35 | 90 | 45 |  | 200 | |
| Ind3 | 1.65 | 210 | 45 |  | 50 | |
| Ind4 | 1.50 | 150 | 45 |  | 50 | |
| Ind5 | 1.40 | 150 | 45 |  | 150 | |
| Ind6 | 1.40 | 150 | 45 |  | 150 | |
| Ind9* | 1.30 | 90 | 550 |  | 0 | |
| Ind10 | 1.60 | 210 | 45 |  | 50 | |
| Ind11 | 1.60 | 210 | 45 |  | 100 | |
| Ind12 | 1.50 | 150 | 45 |  | 100 | |
|  |  |  |  |  |  | |
| ^*^For all mice, the air puff duration for responding after the stop signal was set to 20 ms and timeout for responding after the stop signal was 45 s except Ind 9. For Ind 9, puff duration was set to 80 ms and responding after the stop signal was set to 550 s to ensure stable stopping performance. | | | | | |  |

### **Probe Sessions**

At the probe session stage, stop signal delays were introduced into stop signal trials, so that stop signals occurred at specific time points after the initiation of a trial. Stop signal delays were calculated according to the previous day´s mean reaction time for each animal. Three different delays were set: mean reaction time minus 75, 150 or 300 ms. Table S3 shows mean reaction times and standard deviation of go reaction times during the probe session for each individual as well as pooled data for all individuals. However, in contrast to previous studies, in which the delays were fixed at the beginning of the probe sessions according to the previously recorded mean reaction times, in the present study they were adjusted daily to reflect dynamic changes. This approach was chosen to prevent mice from adapting to the stop signal delay by slowing down or speeding up their response. Such adaptations would have affected the observed results as slowing down a response would give animals more time to stop an initiated response after the stop signal, while speeding up their responses would increase the chance of responding before the stop signal occurred. If a mouse made the nose poke to the middle port before the stop signal was given, the mouse was rewarded (as in a typical go trial). Importantly, for our analysis such a “response before the stop signal is given” or “early trial” was scored as a valid non-inhibited response during a stop trial and the data point was included in the inhibition curve. This was done to prevent a skew in the non-inhibited distribution: According to the two-horse race model, those quickly completed trials are still part of the left tail of the reaction time distribution (Mayse et al, 2014). Therefore, they should not be excluded from the calculations. Probe sessions lasted ten days for each mouse and stop signal reaction times were calculated as previously described (Logan et al., 1984).

| **Table S3.** Mean reaction times and standard deviations of go reaction times during probe trials | | | | |  |
| --- | --- | --- | --- | --- | --- |
| Individual | Mean Reaction Time  [ms] | Standard deviation  [ms] | | |  |
| Ind1 | 946 | 208 | | |  |
| Ind2 | 838 | 166 | | |  |
| Ind3 | 945 | 210 | | |  |
| Ind4 | 908 | 173 | | |  |
| Ind5 | 829 | 165 | | |  |
| Ind6 | 843 | 140 | | |  |
| Ind9 | 896 | 165 | | |  |
| Ind10 | 1032 | 213 | | |  |
| Ind11 | 1055 | 204 | | |  |
| Ind12 | 937 | 200 | | |  |
| Pooled | 921 | 200 | | |  |
|  |  |  |  |  | |

### **Two Horse Race Model of Response Inhibition**

According to the two horse race model, during a stop trial, the actions of completing the go reaction (i.e. second nose poke to the middle port) and stopping the go reaction are racing each other. The outcome of the stop trial (inhibited or non-inhibited) depends on whichever process finishes first. The model assumes that the two processes are independent and therefore do not interfere with each other. As the two processes are independent, the go reactions during the stop signal trials are expected to be same as during go trials. However, if the go responses are slower than the stop response (represented as thick line in Fig. S1), responses would be inhibited. The duration required for stopping is the summation of the given stop signal delay after a “go” signal (i.e. first nose poke to the left port) and stop signal reaction time which is assumed as constant (Fig. S1).

Within this theoretical framework, the stop signal reaction time is estimated using the go reaction time distribution and the observed probability of inhibition for a particular stop signal delay during stop trials. First, the go reaction time coinciding with the stop response time is determined to calculate the stop signal reaction time which corresponds to the percentile calculated as 100 * probability of responding. After this go reaction time is established, the stop signal delay is subtracted from this reaction time to calculate stop signal reaction time (Fig. S1).

According to this model, if individual mice differ only in the mean reaction time (here peak of go reaction distributions) but show the same variance in their go reaction times, setting the stop signal delays relative to the mean reaction time would result in the same inhibition rates for each delay (Fig. S1B). Differences in the variance of the go reaction times on the other hand would affect the steepness of the inhibition curves: The lesser the variance, the steeper the inhibition curve.


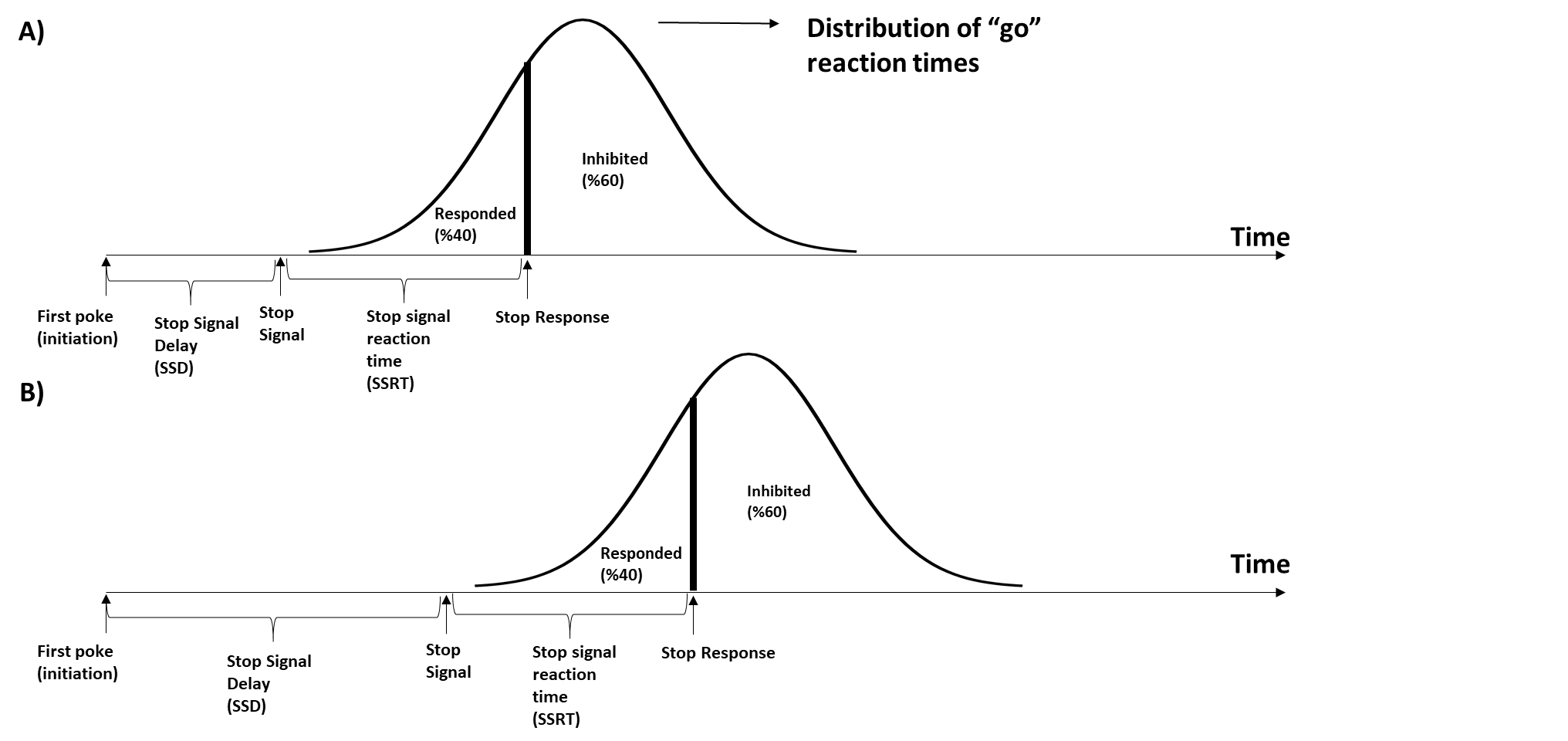


**Fig S1.** Visual representation of the two horse race model. A) Following the go signal (in our study the intrinsic signal of the first nose poke to left port) the go response is initiated. In a stop trial, if the stop signal occurs after a stop signal delay, according to the model the stop response is initiated independent of the go response and these two processes start racing each other. The outcome of the trial depends on whichever process finishes first. If stopping is completed first the go response is inhibited and vice versa. The total time required to stop is a summation of the stop signal delay and stop signal reaction time which is assumed as a constant. The go reaction times show a distribution which is assumed to be the same for go and stop signal trials. Any go responses slower than the time required for stopping (stop response time indicated by thick vertical line) would be inhibited. Therefore, for each stop signal delay, there is a stop response time that corresponds to the probability of responding (1- inhibition). To calculate the stop signal reaction time (SSRT), the stop signal delay is subtracted from the stop response time. B) If two individuals have the same SSRTs but different peaks of the go reaction time distribution (mean reaction time), setting stop signal delays relative to the peaks of the distribution (e.g 150 ms before the individual mean reaction time) will result in the same amount of inhibition as long as the distributions have the same variance (spread of the curve).

### **Data analysis**

### **Correction of the calculated inhibition probabilities relative to omission errors during go trials**

During go trials, mice occasionally fail to complete an initiated trial (omission), either voluntary or for example due to distraction. The same behavior might have occurred during stop trials, in which case the response would wrongly be counted as a successful inhibition. Such an approach would inflate the estimated inhibition probabilities. Therefore, the success rate was corrected for an assumed frequency of omissions according to a formula adopted from Tannock et al., 1989 and Solanto et al., 2001: corrected inhibition rate = observed inhibition rate – omission rate / (1 – omission rate). The results reflect the probability of inhibitions for those trials in which a go response would have been completed in the absence of the stop signal.

### **Inhibition Curves and Stop signal reaction time (SSRT) calculations**

As stop signal delays were set dynamically according to the mean go reaction time of the previous day, stop signal delays varied slightly from day to day. For example, if a mouse’s mean reaction time was 910 ms during the last training day, the delay was set to 610 ms for the stop signal to occur 300 ms before the mRT during the first day of probe sessions. If the animal’s mean reaction time increased to 950 ms during the first probe trial day, for the following day the delay had to be adjusted to 650 ms for the stop signal to occur 300 ms before the adjusted mRT the next day. To simplify SSRT calculations and the construction of inhibition curves, the inhibition data for a particular delay category (300, 150, or 75 ms before mRT) was pooled across days under the assumption that the stop signal delays were given exactly 300, 150 and 75 ms before the grand mean of go reaction times during probe sessions. For example, if an animal had 930 ms as its grand mean of go reaction times, the stop signal delay “300 ms before mRT” was taken as 630 ms regardless of the slight variation in the daily delays given.

The results were also analyzed using two additional approaches: (a) The number of trials per day (i.e. per dynamically adjusted individual reaction time) was taken into account. (b) SSRTs and inhibition values were first calculated for each day. Inhibition curves were then constructed using these daily values and daily SSRTs were averaged across days. These results can be found in the supplementary results section (Fig. S6 and S7).

(a) Weighted average delays were calculated for each delay category (300, 150, or 75 ms before mRT) by multiplying the adjusted delay with the number of stop trials on that day and building the sum across all probe days. The sum was then divided by the total number of stop trials across all probe days for the particular delay category:

*Weighted average delay* ***=*** *(number of stop trials_1_*given_delay_1_+ number of stop trials_2_*given_delay_2_ … number of stop trials_10_*given_delay_10_) /number of stop trials_total_*

For example, if the delay for “300 ms before mRT” was 610 ms for the first day of probe trials and the animal received nine stop signal trials for this delay and the next day the delay was 650 ms and the animal received 11 stop signal trials, then 610 ms would be multiplied by nine and 650 ms would be multiplied by 11. If there were only 2 probe session days, the sum would be calculated across those two days and divided by the total number of stop trials in this delay category: (610 ms * 9 + 650 ms * 11) / (9 + 11) = 632 ms.

(b) In this second approach, SSRTs were first obtained for each day and then averaged across all days. Inhibition curves were constructed based on inhibition values obtained from each independent day. However, it is worth noting, that if for a particular stop signal delay an individual successfully inhibits all stop signal trials (corrected inhibition rate = 1) or shows a corrected inhibition rate of zero or below, it is not possible to estimate the SSRT. As the amount of available data for each stop signal delay is reduced by first analyzing data from each mouse separately for each day, the likelihood of randomly obtaining corrected inhibition rates equal 1 or ≤ 0 increased. In addition to that, it was also not possible to fit corrected daily inhibition rates below zero to the inhibition curve. Unusable values were excluded from analysis without any replacement.

### **Stop signal reaction time (SSRT) calculations**

To calculate the stop signal reaction times for each individual, we rank ordered reaction times from go trials during all the probe session for each individual. Then for each different stop signal delay duration, the nth reaction time was determined where n is the total number of completed go trials multiplied by the probability of responding (1 – corrected inhibition rate). Afterwards, we subtracted the stop signal delay from this nth mean reaction time to obtain an SSRT estimate. After obtaining three SSRT estimates (one for each delay), we averaged those estimates to obtain the final estimate for the SSRT.

### **Theoretical Inhibition Curves**

To examine how well the observed inhibition values matched with theoretical predictions from the two horse race model, we constructed theoretical curves based on parameters obtained from experimental data. For this purpose, we assumed a normal distribution of go reaction times. Based on this assumption for each mouse we used the grand mean as well as the standard deviation of all go reaction times during all probe session trials (Table S3) for our theoretical calculations. To obtain stop response times, we used our SSRT estimates as well as stop signal delays set between 350 and 0 ms (1 ms increments) before the mean reaction time. Next, the rate of responding was calculated for each delay using the cumulative probability distribution of the normal distribution (up until stop response time). Lastly, the inhibition rate was calculated as 1 – response rate.

# **References**

Logan, G. D., Cowan, W. B., & Davis, K. A. (1984). On the ability to inhibit simple and choice reaction time responses: A model and a method. *Journal of Experimental Psychology: Human Perception and Performance*, *10*(2). https://doi.org/10.1037/0096-1523.10.2.276

Mayse, J. D., Nelson, G. M., Park, P., Gallagher, M., & Lin, S. C. (2014). Proactive and reactive inhibitory control in rats. *Frontiers in Neuroscience*, *8 MAY*. https://doi.org/10.3389/fnins.2014.00104

Solanto, M. v., Abikoff, H., Sonuga-Barke, E., Schachar, R., Logan, G. D., Wigal, T., Hechtman, L., Hinshaw, S., & Turkel, E. (2001). The ecological validity of delay aversion and response inhibition as measures of impulsivity in AD/HD: A supplement to the NIMH multimodal treatment study of AD/HD. *Journal of Abnormal Child Psychology*, *29*(3). https://doi.org/10.1023/A:1010329714819

Tannock, R., Schachar, R. J., Carr, R. P., Chajczyk, D., & Logan, G. D. (1989). Effects of methylphenidate on inhibitory control in hyperactive children. *Journal of Abnormal Child Psychology*, *17*(5). https://doi.org/10.1007/BF00916508

Winter, Y., & Schaefers, A. T. U. (2011). A sorting system with automated gates permits individual operant experiments with mice from a social home cage. *Journal of Neuroscience Methods*, *196*(2). https://doi.org/10.1016/j.jneumeth.2011.01.017
