# Supplementary material for: The Stop Signal Task for Measuring Behavioral Inhibition in Mice With Increased Sensitivity and High-Throughput Operation
Source: Front Behav Neurosci. 2021 Dec 9;15:777767. doi: 10.3389/fnbeh.2021.777767 (PMC8696275; doi:10.3389/fnbeh.2021.777767)
Supplement: Supplementary file 2 [file Data_Sheet_2.docx]

# **Supplementary results**

**
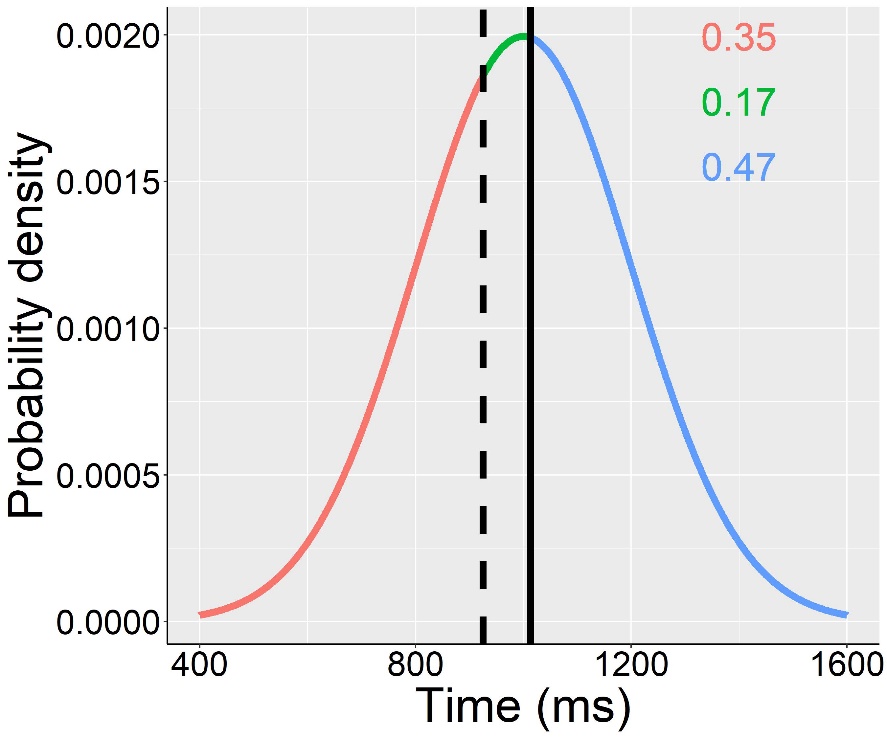
**

**Fig S1.** An example for the outcome probabilities of a stop according to the two horse race model using the following parameters: mean of go reaction times = 1000 ms, standard deviation of go reaction times = 200 ms, stop signal reaction time = 88 ms and stop signal delay = 925 ms (75 ms before mean reaction time). The distribution of go reaction times is assumed to be normal. The dashed line indicates the time of the stop signal presentation and the solid line indicates the time required for the stopping (the summation of the stop signal delay and the stop signal reaction time). The probability density curve is divided into three with the lines to show the probabilities of outcomes: early response before the stop signal (red), response after the stop signal (green) and inhibited response (blue). The expected proportions of those outcomes (as cumulative probabilities) are shown in the left top corner (red: early response, green: response after the stop signal and blue: inhibited response). In this example, excluding early trials would lead to a drastic overestimation of behavioral inhibition (0.73 [0.47/0.64] instead of 0.47). As a consequence, such an overestimation of behavioral inhibition would lead to an underestimate of the SSRT.


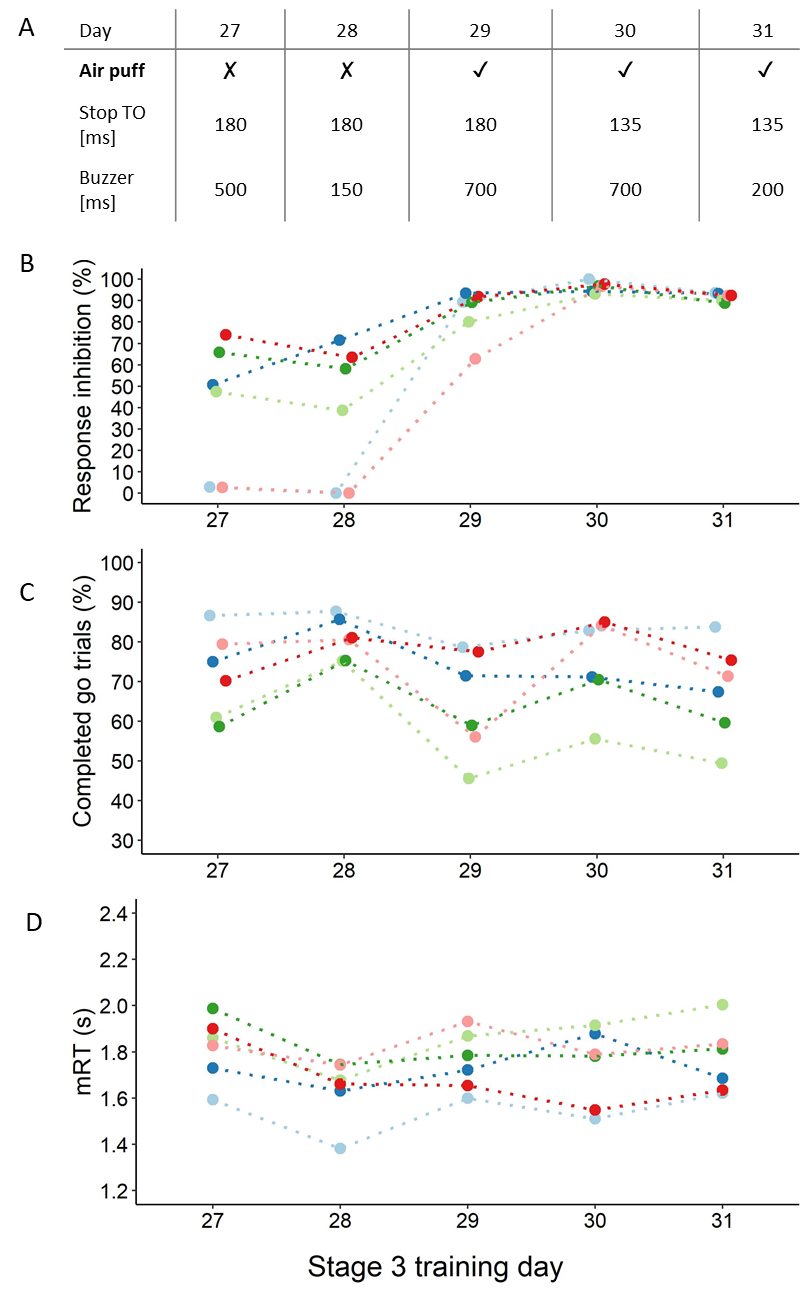


**Fig S2.** Comparison of performance before and after the addition of a negative reinforcer on failed stop trials during a pilot experiment. Six female mice had been trained for 28 days on the third training stage under various variable settings without reaching good response inhibition levels. Following a two day training break, an air puff was used as a negative reinforcer on training days 29-31. For the depicted data, the following variables were kept stable across all days: limited hold period during go trials (2.7 s), refrain time period during stop trials (2.7 s) as well as the go trial timeout (45 s). In addition, and in contrast to the main study, the right port was used as a response port and the middle port as a reward port during the depicted time period. (A) Variables that were changed were the presence or absence of an air puff (100 ms, no delay), the length of the stop trial timeout (stop TO) and the buzzer used as a stop signal. Visual inspection of how changes in these variables affected (B) , response inhibition (C) percentage of completed go trials, and (D) mean reaction time on go trials revealed that the introduction of a negative reinforcer increased response inhibition and decreased response inhibition variability (day 28: median = 48 , interquartile range = 52 vs day 31: median = 92 , interquartile range = 2), while the percentage of completed go trials (day 28: median = 81 , interquartile range = 8 vs day 31: median = 69 , interquartile range = 13), and mean reaction times (day 28: median = 1.67, interquartile range = 0.09 vs day 31: median = 1.75, interquartile range = 0.19) were only slightly affected. In comparison, changes in stop trial timeout and buzzer lengths only had minor effects. Individuals are represented by the same color in all panels. In two cases in which mice had greater omission rates than uncorrected response inhibitions, corrected response inhibitions are shown as zero (instead of negative). Data from n=6 mice.


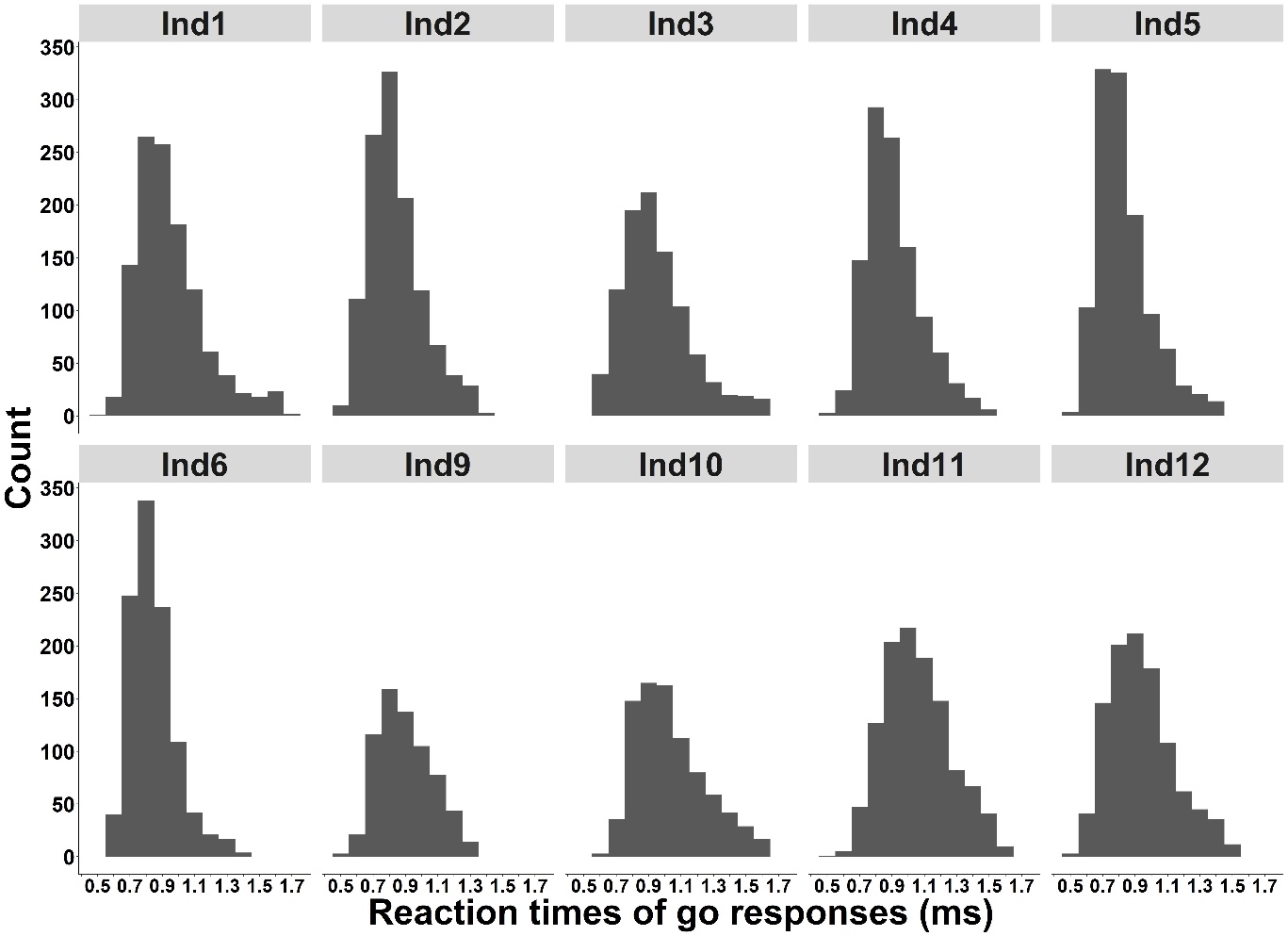


**Fig S3.** Observed distribution of go reaction times during probe trials. The distributions are approximately normal although some of them are slightly right skewed. Therefore, assuming normal distribution for constructing the theoretical inhibition curve was justified. Data from n=10 mice.


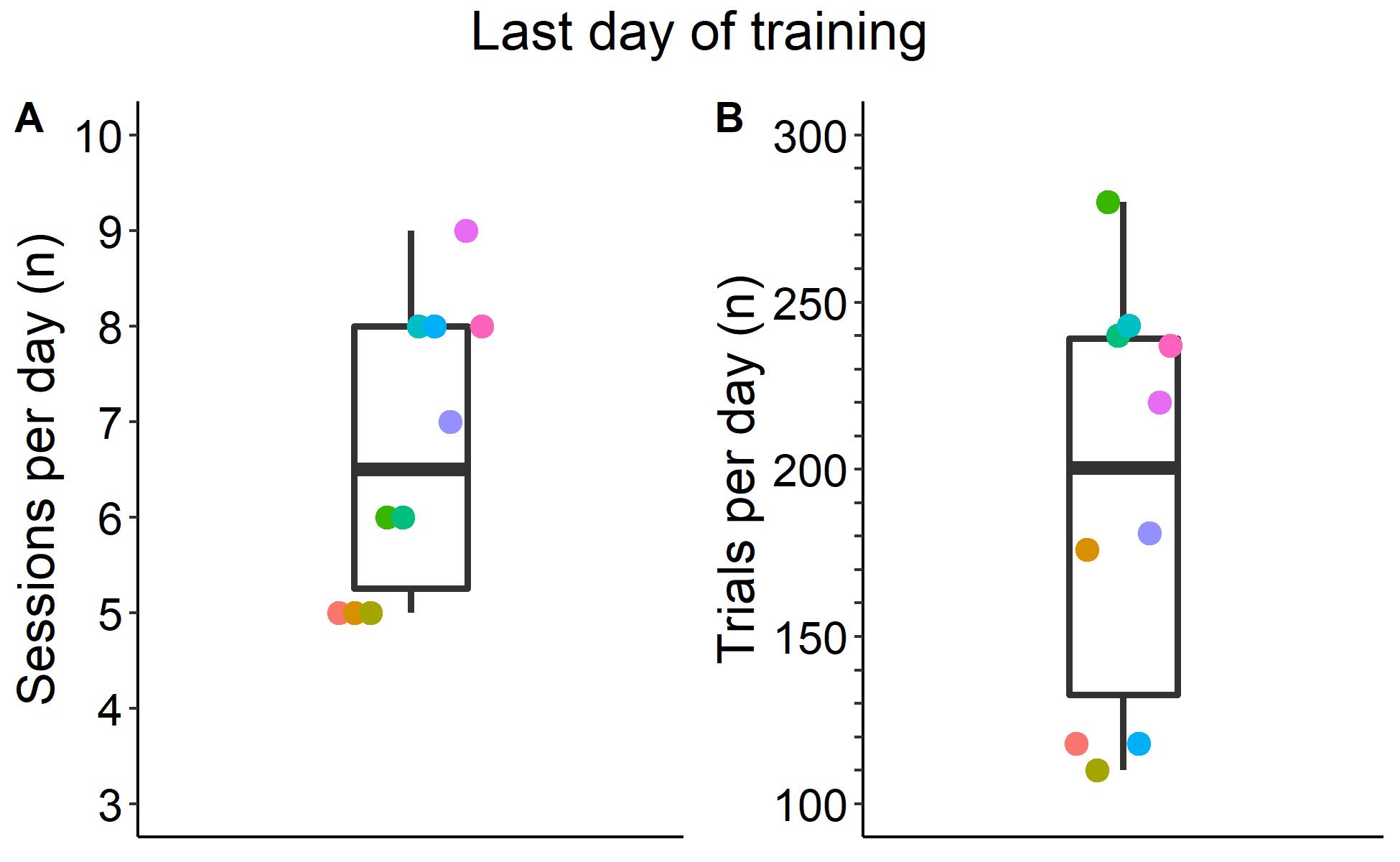


**Fig S4.** Experimental performance during the last day of training. A) Number of sessions ranged from 5 to 9 with a median of 6.5. B) Number of initiated trials ranged from 110 to 280 with a median of 220. Dots show individuals. Box plots show median, 1^st^ and 3^rd^ quartile, and whiskers the 1.5 interquartile range. Data from n=10 mice.


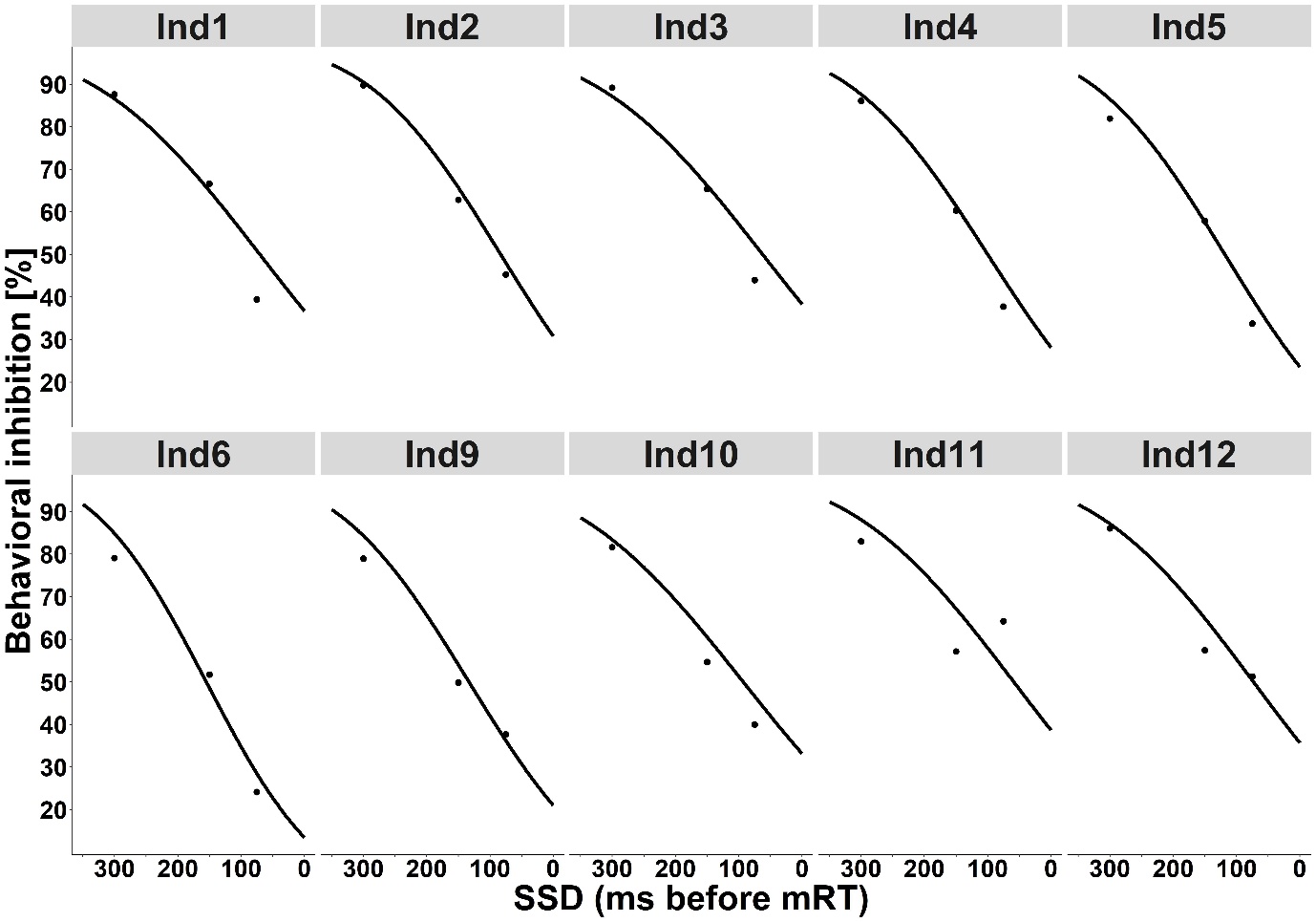


**Fig S5.** Theoretical inhibition curves constructed according to two horse race model for each individual using the experimentally observed parameters (assuming normal distribution of reaction times). The dots show the experimentally observed inhibition values. The observed inhibition values fit reasonably well to the theoretical curves. Data from n=10 mice. SSD stop signal delay; mRT mean reaction time.

**
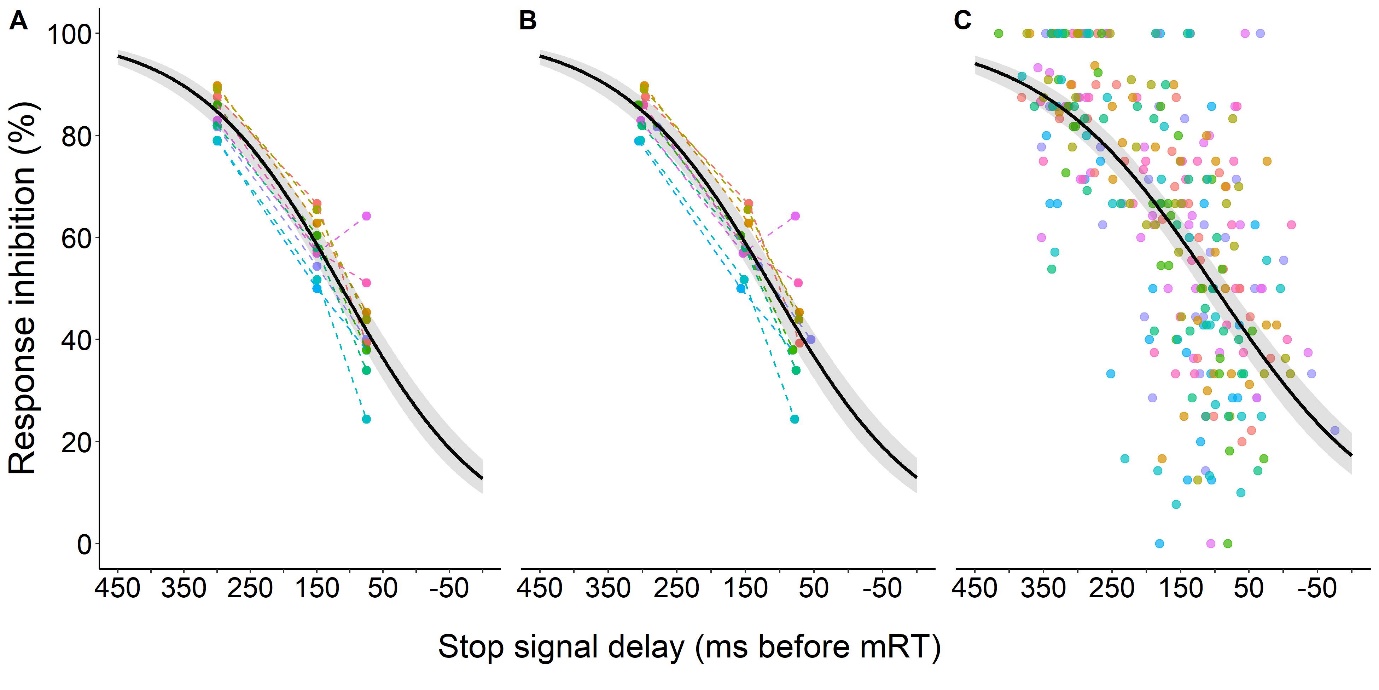
**

**Fig S6.** Inhibition curves based on three different analysis procedures (compare data analysis section in supplementary materials and methods). Individuals in all three panels have the same color. A) Inhibition curve constructed by pooling the stop trials and assuming the delay was given relative to the grand mean of the go reaction times, for example, if the grand mean of the go reaction time was 810 ms, then the delay “150 ms before mRT” was taken as 760 ms. B) Inhibition curve constructed based on weighted average delays in which the different number of stop signal trials per day was taken into account C) Inhibition curve constructed by fitting an inhibition curve for each day. Data from n=10 mice.


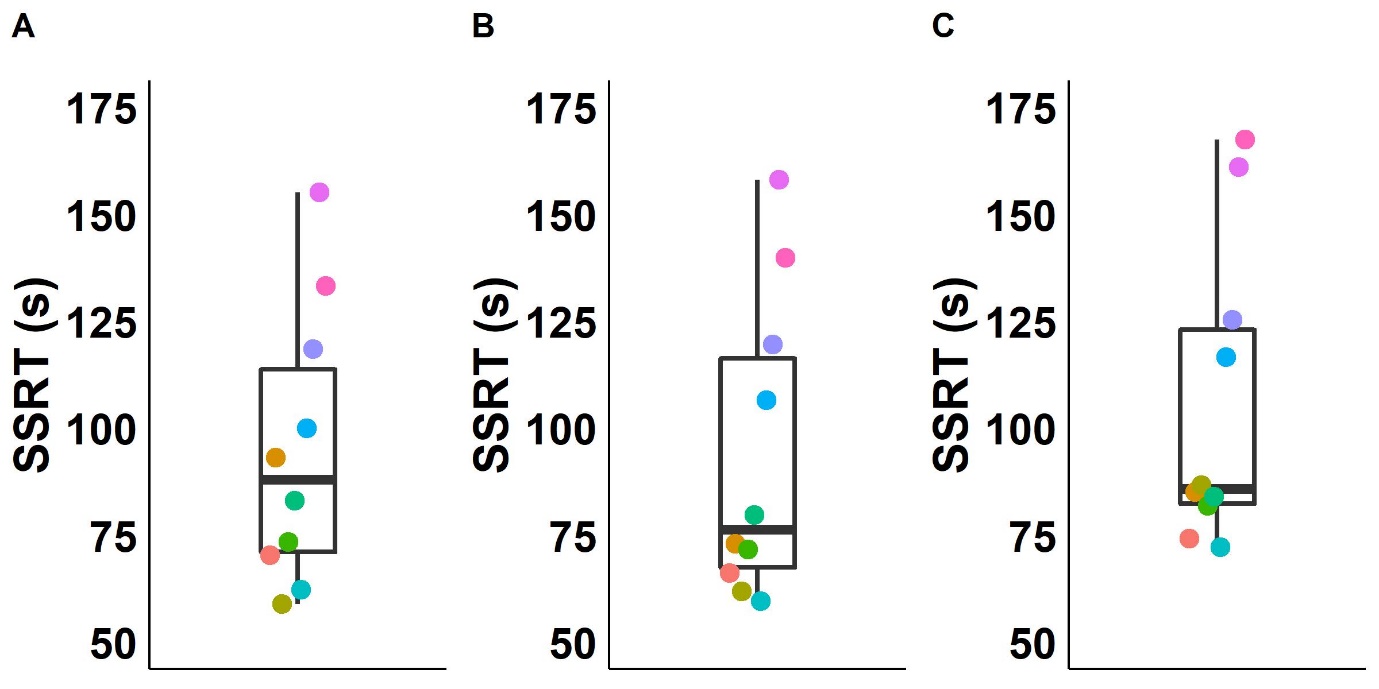


**Fig S7.** Estimated SSRTs based on three different analysis procedures (compare data analysis section in supplementary materials and methods). The three results did not differ substantially from each other. Same colored dots represent the same individuals in all of three panels. A) SSRTs estimated by pooling the stop trials together and assuming the delay was given relative to grand mean of go reaction times B) SSRTs estimated by pooling the stop trials together and calculating the delay as weighted average C) SSRTs estimated by averaging the estimations from each day. Data from n=10 mice. SSRT stop signal reaction time.

### **Inspection of initiation poke durations for successful and omitted go trials**

The initiation poke durations were the durations that mice stayed in the initiation port (the left port) during the trial initiations. To examine if already the initiation poke durations differed between successful “go” trials and omitted “go” trials during the probe sessions, we plotted the normalized histograms of the initiation poke durations for the two conditions for each individual.


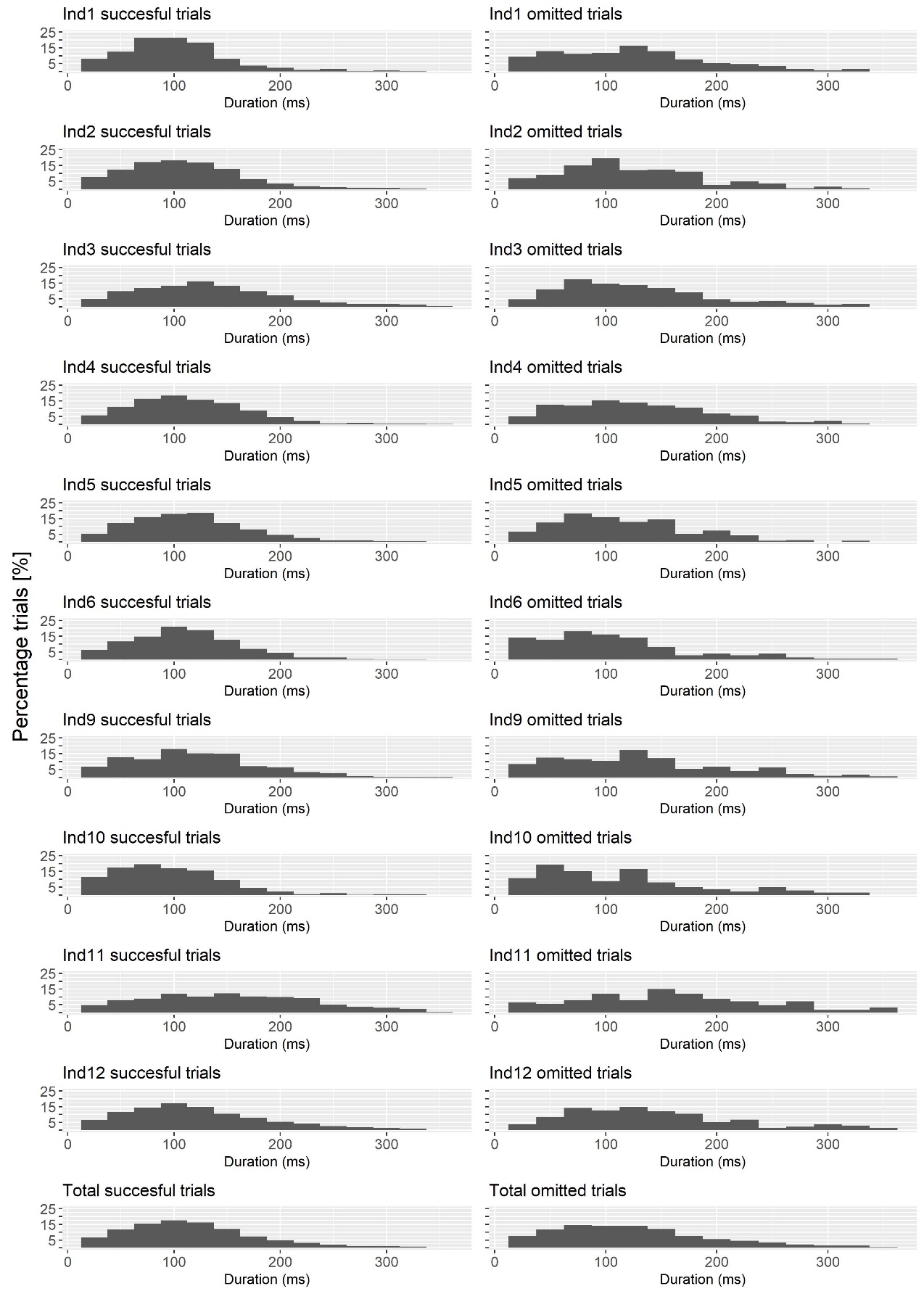


**Fig S8.** Normalized distribution of initiation poke durations (percentage of trials per bin). Across all individuals nose pokes from successful trials were shorter (median = 111 ms) compared to nose pokes from omitted trials (median = 143 ms). Moreover, poke durations preceding successful “go” trials, clustered more around the center of distribution and showed less variance (interquartile range = 80 ms vs 210 ms). Durations longer than 350 ms are not shown (9% of all initiation poke durations) but included in the median.
